# Extended-Release Lithium Treatment for Adolescents with Bipolar Disorder with or Without Comorbid Autism Spectrum Disorder: Protocol of a Longitudinal Prospective Naturalistic Study for the Assessment of Efficacy and Tolerability

**DOI:** 10.3390/jcm13206196

**Published:** 2024-10-17

**Authors:** Gianluca Sesso, Francesca Bargnesi, Giulia Mutti, Stefano Berloffa, Valentina Viglione, Pamela Fantozzi, Greta Tolomei, Fulvio Guccione, Pietro Muratori, Annarita Milone, Gabriele Masi

**Affiliations:** 1 IMT School for Advanced Studies, 55100 Lucca, Italy; gianluca.sesso@fsm.unipi.it; 2 Developmental Psychiatry and Psychopharmacology Unit, IRCCS Stella Maris Foundation, 56128 Pisa, Italy; stefano.berloffa@fsm.unipi.it (S.B.); valentina.viglione@fsm.unipi.it (V.V.); pamela.fantozzi@fsm.unipi.it (P.F.); greta.tolomei@fsm.unipi.it (G.T.); fulvio.guccione@fsm.unipi.it (F.G.); annarita.milone@fsm.unipi.it (A.M.); gabriele.masi@fsm.unipi.it (G.M.); 3 Department of Clinical and Experimental Medicine, University of Pisa, 56122 Pisa, Italy; francesca.bargnesi@fsm.unipi.it (F.B.); giulia.mutti@fsm.unipi.it (G.M.)

**Keywords:** lithium, bipolar disorder, autism spectrum disorder, adolescents, suicidality, emotional dysregulation

## Abstract

**Background**: Lithium is the gold-standard treatment for Bipolar Disorder (BD) in both adults and adolescents, effectively managing mood episodes and reducing suicide risk. While its efficacy in neurotypical youth is well established, its use in adolescents with Autism Spectrum Disorder (ASD) and comorbid BD remains under-researched. Here, we present the protocol for a study aiming to evaluate the efficacy and tolerability of Extended-Release Lithium Salts in treating adolescents with BD and comorbid ASD compared to neurotypical BD patients. **Methods**: This longitudinal prospective naturalistic comparative study will enroll lithium-naïve adolescents aged 12–18 with BD, with or without comorbid ASD, from the Department of Child and Adolescent Psychiatry and Psychopharmacology. Participants will be followed for six months while receiving Extended-Release Lithium Salts treatment. Primary outcomes will include mood instability, suicidality, emotional dysregulation, and aggression, assessed through a range of clinical rating scales and diagnostic tools at baseline, three months, and six months. Secondary outcomes will focus on the safety and tolerability of Extended-Release Lithium Salts, with measures including side effect ratings, physical exams, and laboratory tests. **Results**: We hypothesize that Extended-Release Lithium Salts will demonstrate non-inferiority in treating BD symptoms in adolescents with comorbid ASD compared to those without ASD. **Conclusions**: This study is poised to fill a significant gap in the literature by providing critical data on the use of lithium for adolescents with BD and ASD. Findings will inform clinical practice and future research, potentially guiding more personalized treatment approaches for this complex and vulnerable population.

## 1. Introduction

Lithium is the gold-standard medication for treating Bipolar Disorder (BD), providing significant clinical effectiveness against both depression and mania as well as in preventing mood episodes relapses and reducing the risk of suicide [1,2,3]. For children and adolescents, lithium has been approved by the major regulatory agencies, including the Food and Drug Administration (FDA) and the European Medicines Agency (EMA), for treating BD [4], which shows a high worldwide prevalence up to 3.4% [5]. Nonetheless, less evidence supports the efficacy of lithium in youth compared to adults [6,7,8,9].

A meta-analysis [10] on the treatment of pediatric mania found that lithium effectiveness was unclear, due to the lack of controlled studies at the time, with promising outcomes for second-generation antipsychotics (SGAs). A more recent review [2] found, instead, a level 1b of evidence for lithium on mania in pediatric BD (e.g., [7,11]), in line with the findings of an even more recent systematic review [12] suggesting that lithium is an effective and well-tolerated treatment for some forms of pediatric mania. Indeed, lithium is inferior in efficacy to Risperidone in prepubertal youths diagnosed with mania or mixed episodes and comorbid ADHD [12], while there is a lack of evidence concerning its efficacy and tolerability as acute treatment for manic symptoms in adolescents, and important clinical issues still remain unaddressed.

Less conclusive evidence is available for depression, with suggestive studies providing a level 2b of evidence (e.g., [13,14]). Other long-term studies support the use of lithium in relapse prevention of depressive episodes [6,8]. Interestingly, recent findings from large longitudinal studies suggest strong clinical benefits of lithium in reducing or preventing suicide risk in youth with BD, but these findings are not yet comprehensive. For instance, in 2019, the COBY study [15] examined more than three hundred youths with BD over ten years and found that patients on lithium had lower suicide attempts rates than those on other mood stabilizers. Similarly, a study conducted on Swedish national registries in 2023 [16] demonstrated that higher use of lithium was related to lower suicide rates compared to other treatments. Parasuicidal behaviors, including non-suicidal self-injury (NSSI), might also benefit from lithium treatment, although evidence is still scarce and based on cross-sectional studies [17,18].

Lithium is known to exert its anti-suicidal effects by preventing relapses of mood disorder, though additional mechanisms could also be considered, especially in youth. Indeed, there is some evidence that lithium reduces aggression and impulsivity, which is a possible mechanism mediating its anti-suicidal properties [1]. Adult patients taking lithium exhibit reduced self-harming and unintentional injury compared to those prescribed with Valproate, Quetiapine, or Olanzapine [19]. Lithium use prevents suicidality by decreasing impulsive aggression in addition to mood stabilization. Moreover, the effectiveness of lithium in preventing suicide is associated with decreased aggression and impulsivity, which are often associated with dysphoric, agitated, and mixed states, particularly vulnerable to suicidal events [20]. Additionally, it has been suggested that Lithium may promote its anti-suicidal properties via reinforcing top-down brakes of impulsive actions, and even natural lithium intake in drinking water can influence impulsivity and suicidality [21]. In line with this association, several studies found that lithium is effective in reducing impulsive aggression and temper outbursts in severely violent and hostile CD youth, being significantly superior to placebo [22] either in monotherapy or in add-on with atypical antipsychotics [23].

Lithium also remains commonly used for different psychiatric conditions in both adults and youths, either in or not in comorbidity with BD [2]. For instance, evidence is available for its use in Severe Mood Dysregulation [24], Major Depression as an augmenting medication [25,26], Conduct Disorder and aggression [22,27], Kleine–Levin Syndrome [28] and Fragile X Syndrome [29]. Within the context of neurodevelopmental disorders, lithium is warranted to minimize the risk of manic switch as well as suicidal and non-suicidal self-injurious behavior in ADHD youths with comorbid BD, especially when stimulants are prescribed [30,31,32]. Unfortunately, the use of lithium in people with Autism Spectrum Disorders (ASD) is still poorly investigated.

The association between ASD and BD in adult subjects has been repeatedly confirmed, and lithium has been suggested as the first treatment option for high-functioning ASD with comorbid BD [33]. Similarly, children and adolescents with ASD have high rates of comorbid BD, up to 4–7% in clinical cohorts [34,35], and often experience irritability, aggression, self-injury, and tantrums, as well as subclinical manic symptoms including elation, expansive mood, and racing thoughts [32]. Also, it is increasingly recognized that comorbid BD significantly contributes to the morbidity associated with ASD [36], although the communication difficulties and the frequently low intellectual functioning complicate the identification of comorbid clinical pictures in this population [37]. To further increase this clinical complexity, BD in ASD is often characterized by atypical features, such as irritability and behavioral disturbances, which are frequently associated with several other potentially comorbid conditions, including, above all, ADHD [37,38]. Finally, suicidal behaviors are also frequently observed in ASD-BD patients and may confer a greater risk of hospitalization [39,40] since ASD is an independent risk factor for suicide, with a probability to die by suicide which is ten times greater than in the general population [41].

Despite these considerations, research so far has only scarcely targeted this comorbid population to define guidelines for the clinical management of associated morbidity. Pharmacological treatment options available for ASD with comorbid BD are not directed to the core symptoms of the disorder but rather target challenging behaviors associated with comorbidities [32]. Second-generation antipsychotics (SGAs)—i.e., Risperidone and Aripiprazole—are currently approved by FDA and EMA for treating irritability and aggression in ASD youths [42]. Approximately 65% of ASD-BD patients treated with SGAs showed an improvement greater than 30% in irritability, and despite an atypical severity of BD symptoms in ASD youths, the presence of this comorbidity does not impact the relative rate of response [43]. However, given the high risk of SGA-related side effects, including weight gain and metabolic disorders, increased serum prolactin, and sedation, antipsychotics should be used cautiously [32].

On the other hand, lithium has been rarely studied in children with ASD, despite its established efficacy in controlling mood disorder symptoms in the neurotypical population, and controlled trials supporting its efficacy for mood instability, impulsivity, and cyclicity in ASD subjects are lacking. However, some case reports [44,45,46] and two retrospective chart reviews are available for this purpose [47]. In the first of these latter [48], 43% of ASD patients who received lithium improved after treatment, along with 71% of those who had two or more pretreatment mood disorder symptoms including mania and euphoria. In the second [49], 73.7% of patients with ASD experienced significant clinical improvement after add-on lithium salts to their treatment regimen, thus representing a reliable alternative to antipsychotics and other psychotropic medications.

Thus, the present article will present the research protocol for a longitudinal prospective study aiming to assess the efficacy and tolerability of Extended-Release Lithium Salts treatment in adolescents with ASD and comorbid BD versus neurotypical BD patients. This naturalistic comparative study will specifically focus on BD-related morbidity often associated with ASD, including suicidality, non-suicidal self-injury, irritability/emotional dysregulation (ED), and aggression in a longitudinal sample of referred youths with BD with and without ASD. In the absence of previous research on this topic, we hypothesize the non-inferiority of Extended-Release Lithium Salts in adolescent patients with BD and ASD compared with those without comorbid ASD.

## 2. Materials and Methods

### 2.1. Study Design

This is a longitudinal prospective naturalistic comparative intervention study including BD youths with or without comorbid ASD, enrolled at the Department of Child and Adolescent Psychiatry and Psychopharmacology of our third-level hospital. This study will be conducted in accordance with the Declaration of Helsinki and approved by the Regional Ethics Committee for Clinical Trials of Tuscany (Pediatric Ethics Committee at Meyer Children’ Hospital of Florence). The aim of the study will be to evaluate the efficacy (primary aim) and tolerability (secondary aim) of Extended-Release Lithium Salts treatment in lithium-naïve adolescent patients with BD with versus without comorbid ASD. The STROBE (Strengthening the Reporting of Observational studies in Epidemiology) Statement Guidelines—Checklist for cohort studies will be followed to ensure an improved quality of reporting the results of our study.

### 2.2. Recruitment

Patients will be consecutively enrolled after assessment at our hospital for eligibility. Those who meet the eligibility criteria listed below will be included following a thorough clinical screening. All participants and their caregivers will be informed about the assessment procedure, and participation in the study will be voluntary. Recruited patients and their parents will be asked to provide their signed informed consent. Additionally, patients will be allowed to withdraw their consent any time during the study.

Inclusion criteria will be as follows: lithium-naïve adolescent inpatients or outpatients aged 12 to 18 years; informed consent obtained from the patient and both caregivers; diagnosis of Bipolar or Related Disorders (Bipolar Disorder type I (BD-I), Bipolar Disorder type II (BD-II), Cyclothymic Disorder, Other Specified or Unspecified Bipolar Disorder) with or without a diagnosis of Autism Spectrum Disorder according to the Diagnostic and Statistical Manual of Mental Disorders, Fifth Edition, Text Revision (DSM-5-TR); cognitive functioning within the borderline to normal range (i.e., Full-Scale Intelligence Quotient (FSIQ) or General Ability Index (GAI) ≥ 70); current presence of Self-Injurious Thoughts and/or Behaviors (SITB) (i.e., including non-suicidal self-injury (NSSI), intentional self-harm, suicide ideation, plans or preparations, or suicide attempts) as assessed through clinical interview (i.e., investigated by administering the Columbia—Suicide Severity Rating Scale); current presence of Emotional Dysregulation (ED) and/or irritability as assessed by means of standardized clinical measures (i.e., Reactivity, Intensity, Polarity, and Stability questionnaire for Youth and/or Affective Reactivity Index above the clinical cutoff). These latter criteria will be considered to ensure a relatively high clinical severity of the included sample.

Exclusion criteria will be as follows: patients under ongoing or previous treatment with lithium in any formulation; novel or unstable (i.e., modified posology) treatment with any other psychotropic drugs during the last six weeks; presence of intellectual disability; diagnosis of Schizophrenia Spectrum and Other Psychotic Disorders; presence of cannabis- or other substance-induced psychotic symptoms; females during pregnancy and/or breastfeeding; history of neuroleptic malignant syndrome and/or severe neurologic or neurodegenerative conditions; current diagnosis of epilepsy and/or history of seizures; any acute or unstable medical condition that may prevent study participation.

### 2.3. Procedure and Assessment

A matching number of BD patients with and without comorbid ASD will be recruited. The ASD-BD group will be the experimental arm of the study, with the BD-only group as the clinical comparator. Both groups will be followed up for six months after recruitment while receiving treatment with Extended-Release Lithium Salts for clinical indication. Primary and secondary outcome measures will be acquired before treatment initiation (T0), as well as three (T1) and six (T2) months after reaching stable drug dosage (Steady State—SS). At T0, patients will be screened for eligibility and carefully evaluated by means of the following diagnostic tools: the semi-structured DSM-5-based clinical interview Kiddie Schedule for Affective Disorders and Schizophrenia—Present and Lifetime version (K-SADS-PL); the Wechsler Intelligence Scale for Children—fourth edition (WISC-IV); the Autism Diagnostic Observation Schedule—second edition (ADOS-2); and the Autism Diagnostic Interview—Revised (ADI-R).

At each timepoint (T0, T1, and T2), patients will be thoroughly assessed as for clinical routine. The following rating scales will be filled in by clinicians based on parents’ and caregivers’ reports: the Clinical Global Impression (CGI) scale scores and the Children Global Assessment Scale (CGAS) scores. Patients will be also administered the following clinical interviews: the Children Depression Rating Scale (CDRS); the Young Mania Rating Scale (YMRS); the Columbia—Suicide Severity Rating Scale (C-SSRS); the Deliberate Self-Harm Inventory (DSHI); Reactivity, Intensity, Polarity and Stability questionnaire for Youth (RIPoSt-Y). Caregivers will be administered the following clinical rating scales: the Modified Overt Aggression Scale (MOAS) and the Affective Reactivity Index (ARI).

At each timepoint (T0, T1, and T2), patients will be also assessed for safety and tolerability of Extended-Release Lithium Salts treatment according to following procedures. They will be administered the Lithium Side Effects Rating Scale (LiSERS) to evaluate frequency and severity of side effects. Major adverse events will be monitored and reported to the Italian Medicines Agency according to the clinical routine. They will undergo a full clinical assessment including history update for adverse events, physical and neurological examination, and acquisition of physical and vital parameters (i.e., weight, height, blood pressure, heart frequency), as well as blood sampling and EKG. Blood sampling will be performed the day of each visit in the morning through venipuncture. Blood tests will include lithium plasma levels, complete blood count, glucose, triglycerides, cholesterol, blood urea nitrogen (BUN), creatinine, serum electrolytes, liver enzymes, creatine-phosphokinase (CPK), amylase, fT3, fT4, and thyroid-stimulating hormone (TSH). Routine urinalysis will be also performed. Resting EKG will be performed before the visit and examined by expert cardiologists to provide the following parameters: heart frequency, P wave duration, PR interval duration, QRS interval duration, and QT and corrected QT interval duration.

### 2.4. Clinical Measures

The following clinical measures will be administered as detailed above:

- CGI: this item evaluates the severity of the disease based upon the clinician’s judgement and expertise by coding it on a 7-point scale from 1 = “normal” to 7 = extremely severe”;
- CGAS: this item provides a measure of the degree of functional impairment due to the disease, in terms of social, familiar and school functioning, by coding it on a 100-point score, with values greater than 60 indicating significant clinical impairment;
- CDRS: this 17-item rating scale assesses the severity of depressive symptomatology on a total score ranging from 17 to 113 based on single items rated from 1 to 5 or 7;
- YMRS: this frequently used rating scale aims to assess manic symptoms in youth and is composed of 11 items subjectively rated by the clinicians based on the last 48 h before the interview;
- C-SSRS: this tool is a clinical interview that comprehensively assesses suicidal thoughts and behaviors by investigating their presence, severity, and frequency, including specific sections dedicated to the severity of suicidal ideation, its intensity, the frequency of occurrence of suicide attempts, both failed and interrupted, and self-injurious behaviors, and the lethality of completed suicide attempts;
- DSHI: this self-reported questionnaire is composed of 17 items exploring the presence of several different types of non-suicidal self-injurious behaviors, by specifying, for each type, their frequency and intensity;
- RIPoSt-Y: this self-rated measure evaluates the presence of ED in youth based on 31 items distributed across three different subscales, including Affective Instability, Emotional Reactivity, and Interpersonal Sensitivity, by providing, for each, a clinical cutoff;
- MOAS: this interview is administered to parents or caregivers to measure four types of aggression, including verbal aggression, aggression towards objects, and self-directed and others-directed aggression, by providing a 4-level severity score;
- ARI: this parent-reported questionnaire evaluates the presence of irritability in the last six months and is composed of six items rated from 0 to 2 and a seventh item related to the degree of functional impairment.

### 2.5. Sample Size Calculation

Sample size has been estimated using G*Power software (version 3.1.9.7), considering the clinical rating scales described above as outcome measures of the study. Based on previous literature on BD youths treated with lithium [50], the YMRS score was the best available measure to determine the effect size that would be worth testing and thus was taken as the reference. The calculation was based on a repeated measures test between two independent mean differences of the main outcome, establishing the error level at 0.05, power at 0.95, and effect size at 1.06. Based on this setting, a sample size of 40 subjects (20 patients with ASD-BD and 20 BD-only controls) was obtained. However, since a dropout rate of 20% is expected after 6 months of follow-up based on our previous studies, 5 additional participants per group will be recruited, leading to a total number of 50 subjects as the final sample size (25 patients with ASD-BD and 25 BD-only controls).

### 2.6. Statistical Analyses

Statistical analysis will be performed using RStudio^®^ (version 1.3.1093, RStudio, PBC, Boston, MA, USA). Non-parametric tests for repeated measures (e.g., Wilcoxon signed rank test) will be used to analyze the psychiatric assessment score variations of the clinical rating scales described above. Additionally, multiple linear regression models will be performed to detect the effect of the ASD diagnosis on the variations on patients’ clinical scores. Patients’ age and gender, ongoing treatment with other psychotropic drugs, and lithium plasma levels will be considered as covariates in multivariate analyses to account for possible specific effects.

## 3. Discussion

The present study seeks to address a significant gap in the literature regarding the efficacy and tolerability of Extended-Release Lithium Salts in adolescents with Bipolar Disorder and comorbid Autism Spectrum Disorder. Lithium, widely recognized for its effectiveness in treating BD in the neurotypical population [1,2], has been insufficiently studied in individuals with ASD, particularly in youth populations [32,48,49]. The inclusion of Extended-Release Lithium Salts as a treatment modality in this population is a critical step toward understanding its potential benefits and limitations in this specific comorbid condition.

The longitudinal prospective design of the present study allows for a comprehensive assessment of both efficacy and safety, providing valuable insights into the clinical management of BD in ASD. By focusing on key outcome measures such as mood instability, suicidality, irritability, and aggression, which are prevalent in this population [32,41], the study aligns with the clinical challenges often observed in ASD-BD patients. The inclusion of a well-matched control group of neurotypical BD patients, in which lithium efficacy has been already established [1,2], further strengthens the study capacity to discern the specific effects of Extended-Release Lithium Salts in the context of comorbid ASD.

Another strength of the study is its naturalistic approach, which reflects real-world clinical settings and may enhance the generalizability of the findings. Additionally, the thorough assessment procedures, including the use of validated diagnostic tools and clinical rating scales, ensure a robust evaluation of both primary and secondary outcomes. The careful consideration of covariates, such as age, gender, and concurrent psychotropic treatments in the statistical analysis, will allow for a more nuanced understanding of the factors influencing treatment outcomes. Finally, the sample size calculated to achieve sufficient power will provide further statistical robustness.

Limitations of the study could include the non-randomized design and the lack of a control mediation (e.g., other mood stabilizers), as well as the comparison between different lithium formulation (e.g., extended versus immediate-release lithium formulation). These factors limit the generalizability of the results and underscore the need for further randomized controlled trials to confirm our future findings. Nonetheless, the longitudinal prospective naturalistic design of our intervention study will have significant benefits, including those related to its real-world setting, which provides insights into how interventions work in everyday life, enhancing the external validity of the findings.

## 4. Conclusions

This study will have the potential to make a significant contribution to the field. By investigating the efficacy and safety of Extended-Release Lithium Salts in a previously under-investigated population, it may provide critical evidence to guide clinical practice and inform future research. The study findings could pave the way for more personalized treatment approaches for adolescents with BD and comorbid ASD, ultimately improving outcomes for this vulnerable population. Specifically, if the results show that lithium offers advantages over harmful BD-related outcomes in youth with ASD, such as suicidality, while maintaining a manageable side effect profile, our study will assist clinicians in considering this often overlooked treatment option for this highly vulnerable group.

## Data Availability

No new data were created.
